# A Functional Chemiluminescent Probe for in Vivo Imaging of Natural Killer Cell Activity Against Tumours

**DOI:** 10.1002/anie.202011429

**Published:** 2021-02-02

**Authors:** Jamie I. Scott, Sara Gutkin, Ori Green, Emily J. Thompson, Takanori Kitamura, Doron Shabat, Marc Vendrell

**Affiliations:** ^1^ Centre for Inflammation Research The University of Edinburgh 47 Little France Crescent Edinburgh EH16 4TJ UK; ^2^ Tel Aviv University Dpt of Organic Chemistry School of Chemistry, Faculty of Exact Sciences Tel Aviv 69978 Israel; ^3^ MRC Centre for Reproductive Health The University of Edinburgh 47 Little France Crescent Edinburgh EH16 4TJ UK

**Keywords:** activatable probes, cancer, chemiluminescence, immunology, natural killer cells

## Abstract

Natural killer (NK) cells are immune cells that can kill certain types of cancer cells. Adoptive transfer of NK cells represents a promising immunotherapy for malignant tumours; however, there is a lack of methods to validate anti‐tumour activity of NK cells in vivo. Herein, we report a new chemiluminescent probe to image in situ the granzyme B‐mediated killing activity of NK cells against cancer cells. We have optimised a granzyme B‐specific construct using an activatable phenoxydioxetane reporter so that enzymatic cleavage of the probe results in bright chemiluminescence. The probe shows high selectivity for active granzyme B over other proteases and higher signal‐to‐noise ratios than commercial fluorophores. Finally, we demonstrate that the probe can detect NK cell activity in mouse models, being the first chemiluminescent probe for in vivo imaging of NK cell activity in live tumours.

## Graphical abstract

In vivo detection of natural killer (NK) cell activity against tumours was achieved with an activatable chemiluminescent probe containing a granzyme B‐reactive peptide substrate linked to a phenoxydioxetane scaffold through a *p*‐aminobenzyl alcohol linker. The rapid and specific chemiluminescence response of the probe was used to detect NK cell activity against breast cancer cells in living mice.

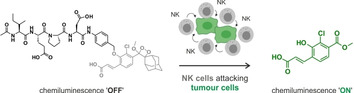

Immunotherapies, including treatments with genetically‐engineered cells or antibodies against immune checkpoint receptors, harness the cytotoxic potential of immune cells and show efficacy for the elimination of tumours.[^1^, ^2^, ^3^, ^4^, ^5^, ^6^] Among these, the administration of natural killer (NK) cells after in vitro expansion has emerged as a promising immunotherapy. NK cells are innate immune cells with a protective role against viral infections and tumours. Clinical trials of adoptive transfer of NK cells have shown positive results in different types of cancer (e.g., acute myeloid leukemia and ovarian cancer)[^7^, ^8^, ^9^, ^10^, ^11^] but there are cases where NK cells are unable to eradicate tumours.^[12]^ Functional imaging probes that could detect the activity of NK cells in tumours would help to identify optimal treatments in a patient‐specific manner.

Upon recognition of target tumour cells, NK cells release cytolytic granzymes into target cells, where they induce apoptosis.[^13^, ^14^, ^15^] The tumour‐killing capacity of NK cells can be monitored in vitro by different methods. The gold standard method is a chromium release assay[^16^, ^17^] where the levels of radioactive ^51^Cr from target cells ‐after lysis by NK cells‐ are measured. These assays have limited sensitivity, safety issues and are unable to detect early apoptosis. Flow cytometry has been used to monitor NK cell cytotoxicity,[^18^, ^19^, ^20^] whilst ELISA tests can quantify the release of cytokines associated with NK cell function.^[21]^ However, these methods have limited spatiotemporal resolution and are incompatible with in vivo imaging. Alternatively, imaging approaches including those relying on activatable probes[^22^, ^23^, ^24^, ^25^, ^26^, ^27^, ^28^, ^29^, ^30^, ^31^] have opened an avenue for the acquisition of functional readouts in living organisms. To date, there are few chemical probes to directly report NK cell activity in tumours in vivo.

Chemiluminescent luminophores are excellent scaffolds for the construction of highly sensitive probes because they do not require incident light. This feature enables detection of low concentrations due to minimal background. Recently, our groups have developed chemiluminescent agents utilising self‐immolative linkers that disassemble spontaneously to “turn on” the emission of quenched chemiluminophores for the detection of proteases^[32]^ and metabolites in preclinical models.[^33^, ^34^] Herein we describe the first chemiluminescent probe to measure the activity of NK cells against cancer cells in vitro and in vivo. Our probe reacts with active granzyme B, a serine protease that NK cells deploy into cancer cells to induce apoptosis. Because the enzymatic activity of granzyme B is directly related to the cell death initiated by NK cells, this probe behaves as a direct reporter of the host's immune cytotoxicity in tumours. We have rationally designed a probe whereby a granzyme B‐specific substrate was coupled to an activatable phenoxydioxetane luminophore via a self‐immolative linker so that chemiluminescence would be only detected in the presence of active granzyme B released by NK cells (Scheme 1).

**Scheme 1:**
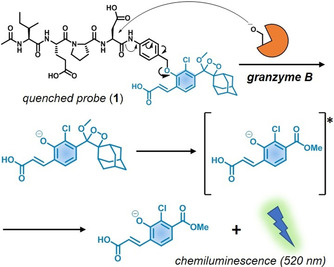
Activation mechanism of the chemiluminescent probe **1**.

We developed the peptide‐based chemiluminescence probe **1** utilising the sequence Ile‐Glu‐Pro‐Asp (IEPD), which was reported by Thornberry et al. as a substrate for granzyme B.^[35]^ Previous reports on the IEPD sequence have shown its reactivity with granzyme B, enabling the development of granzyme B inhibitors as well as radiolabelled imaging probes for positron emission tomography.[^36^, ^37^] Probe **1** is composed of 3 chemical moieties: 1) the granzyme B‐cleavable peptide sequence Ac‐IEPD, 2) a self‐immolative *p*‐aminobenzyl alcohol (PABA) spacer including an electrophilic amide bond to favour enzymatic reactivity, and 3) a phenoxydioxetane moiety that emits chemiluminescence upon cleavage of the construct.

We started the chemical synthesis of probe **1** by preparing the protected IEPD sequence using solid‐phase peptide synthesis^[38]^ (see Supplementary Information for synthetic details). The side chains of aspartic and glutamic acids were protected as allyl esters to isolate the peptide as a free carboxylic acid at the C‐terminus upon cleavage from the 2‐chlorotrityl linker on polystyrene resin (Scheme 2). The carboxylic group of the tetrapeptide was then coupled to the aniline group of PABA using *N*‐ethoxycarbonyl‐2‐ethoxy‐1,2‐dihydroquinoline (EEDQ) to render compound **2**. Importantly, the PABA moiety accomplished two key functions: 1) to introduce a self‐immolative spacer that could be cleaved by granzyme B favouring catalytic efficiency and preventing spontaneous decomposition of the chemiluminescent reporter,^[32]^ 2) to increase the separation between Ac‐IEPD and the phenoxydioxetane moiety and reduce steric hindrance at the cleavage site. Compound **2** was treated with chlorotrimethylsilane and sodium iodide to yield compound **3**, which contained a reactive benzylic group for further substitution. Next, compound **3** was reacted with the acryl‐substituted phenol enol‐ether to give the protected precursor **4**. The allyl esters were deprotected using Pd(PPh_3_)_4_ and dimethyl barbituric acid (DMBA), and the resulting crude reacted with singlet oxygen ‐generated by methylene blue under light irradiation‐ to form the final dioxetane moiety. Purification by HPLC rendered the chemiluminescent probe **1** in >95 % purity with an overall yield around 30 %.

**Scheme 2:**
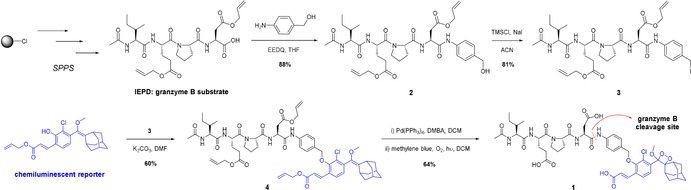
Synthetic scheme for the preparation of the granzyme B‐activatable probe **1**.

With probe **1** in hand, we examined its chemiluminescence readout as a function of time in the absence and presence of active human recombinant (hr)‐granzyme B. As shown in Figure 1, the incubation of probe **1** with the enzyme resulted in peptide cleavage ‐the rate‐limiting step‐ (HPLC analysis in Supplementary Figure 1) and strong chemiluminescence emission around 520 nm within minutes and with very low background signals^[39]^ (Figure 1 a). The rapid response and almost background‐free emission from probe **1** resulted in observed kinetic constants that appear more favourable than those previously reported for granzyme B‐responsive fluorescent constructs (Supplementary Figure 2).[^40^, ^41^, ^42^] Furthermore, we co‐incubated probe **1** and the active enzyme in the presence of the granzyme B inhibitor Ac‐IEPD‐CHO.^[43]^ The inhibitor decreased the chemiluminescence by more than 90 %, confirming that the activation of probe **1** is due to its reactivity with granzyme B (Figure 1 a). Finally, we assessed the selectivity of probe **1** for granzyme B over other proteases (e.g., granzyme A, neutrophil elastase, caspases) as well as biomolecules that might cross‐react with the probe. Notably, probe **1** exhibited 139‐fold chemiluminescence increase in less than 10 minutes after reacting with granzyme B, while we observed much smaller changes in chemiluminescence with other enzymes ‐detectable signals were only found with caspase 8‐ and biomolecules under the same conditions (Figure 1 b). Altogether, these results indicate that probe **1** can rapidly monitor granzyme B activity with good selectivity over other biologically relevant analytes.

**Figure 1:**
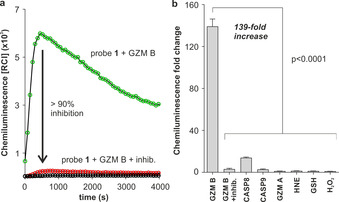
a) Chemiluminescence spectra of probe **1** (100 μm) in aqueous buffer at 37 °C after incubation with hr‐granzyme B (20 nm) (green line) or without enzyme (black line) under the same conditions. Competition experiments with granzyme B inhibitor (Ac‐IEPD‐CHO, 100 μm) are shown in red. Data as mean values (*n*=3). b) Chemiluminescence fold change of probe **1** (100 μm) after incubation at 37 °C with serine proteases (20–40 nm; GZM B: granzyme B, CASP8: caspase 8, CASP9: caspase 9, GZM A: granzyme A, HNE: human neutrophil elastase) and other biomolecules (100 μm: GSH: glutathione, H_2_O_2_: hydrogen peroxide). Data as mean values±SEM (*n*=6–9). P values from ONE‐ANOVA tests with multiple comparisons.

Next, we compared the sensitivity of probe **1** to activatable fluorescent probes featuring the same peptide sequence. The commercially available fluorogenic probe Ac‐IEPD‐AMC contains the 7‐amino‐4‐methylcoumarin moiety, which fluoresces at 450 nm after cleavage by granzyme B (Figure 2 a). We measured the signal‐to‐noise (S/N) ratios of probe **1** and Ac‐IEPD‐AMC after reaction with active granzyme B for 30 minutes. The ratios observed for probe **1** were higher than for Ac‐IEPD‐AMC and achieved S/N values around 100 in less than 10 minutes (Figure 2 b) whereas Ac‐IEPD‐AMC ratios were consistently under 5. Finally, we determined the limit of detection for granzyme B using probe **1** and obtained values around 0.7 nm, asserting the high sensitivity of this chemiluminescent construct (Supplementary Figure 3). Of note, these concentrations are within the dynamic range of extracellular granzyme B reported in clinical samples,[^44^, ^45^, ^46^] which highlights the potential of probe **1** for biomedical applications.

**Figure 2:**
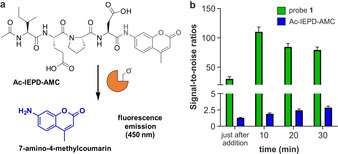
a) Structure and activation mechanism for the fluorogenic probe Ac‐IEPD‐AMC. b) Time‐course signal‐to‐noise ratios after probe **1** (green bars, 100 μm) and Ac‐IEPD‐AMC (blue bars, 25 μm) were independently incubated with hr‐granzyme B (20 nm) in aqueous buffer at 37 °C. Data as mean values±SEM (*n*=6).

Given the excellent in vitro results, we examined whether probe **1** could be used to detect granzyme B‐mediated activity of NK cells against cancer cells under a PhotonImager™ luminescence imaging system. First, we assessed whether the imaging system could detect the signals derived from the reaction of probe **1** and the enzyme. Bright chemiluminescence was observed at increasing concentrations of probe **1**, with the lowest concentrations of probe being detectable in the low micromolar range (Supplementary Figure 4). Next, we prepared in vitro co‐cultures of human NK‐92 cells and the human breast adenocarcinoma cell line MDA‐MB‐231. Given previous reports of NK‐cell‐based cytotoxicity assays,[^47^, ^48^] NK‐92 cells and MDA‐MB‐231 cells were co‐cultured in a 8:1 ratio and in the presence of interleukin‐2 (IL‐2), an essential cytokine for the survival, proliferation and activation of NK‐92 cells.^[49]^ Under these conditions, NK‐92 cells induced apoptosis of MDA‐MB‐231 cancer cells, as shown by fluorescence microscopy using a caspase 3 marker^[50]^ (Supplementary Figure 5). Cells were co‐incubated for different times, followed by the addition of probe **1** and wash‐free image acquisition (Figure 3 a). Probe **1** showed very low emission in media as well as in NK‐92 cells and MDA‐MB‐231 cells alone; however, bright chemiluminescence was detected in the co‐cultures of NK‐92 and MDA‐MB‐231 cells (Figure 3 and Supplementary Figure 6). In line with the anti‐cancer activity of NK cells,^[15]^ we observed that the chemiluminescence of probe **1** increased with the co‐incubation time, with the brightest signals detected after 8 hours (Figure 3 c). Furthermore, we verified that the chemiluminescence signals in the co‐cultures were dependent on granzyme B activity as a significant reduction of the signals was observed upon addition of the Ac‐IEPD‐CHO inhibitor (Supplementary Figure 7). We also used antibody staining to image granzyme B in MDA‐MB‐231 cells before and after co‐culture with NK‐92 cells and found that the levels of intracellular granzyme B were significantly increased after co‐culture (Supplementary Figure 8). This observation suggests that NK‐92 cells can release granzyme B in the proximity of MDA‐MB‐231 cells. Collectively, our results confirm that probe **1** provides a direct optical readout of NK cell activity against tumours via detection of active granzyme B, either in the extracellular milieu or intracellularly in target cancer cells. The speed and simplicity of this protocol may open new opportunities to assess the efficacy of NK cell‐based anti‐cancer treatments in patient biopsies.

**Figure 3:**
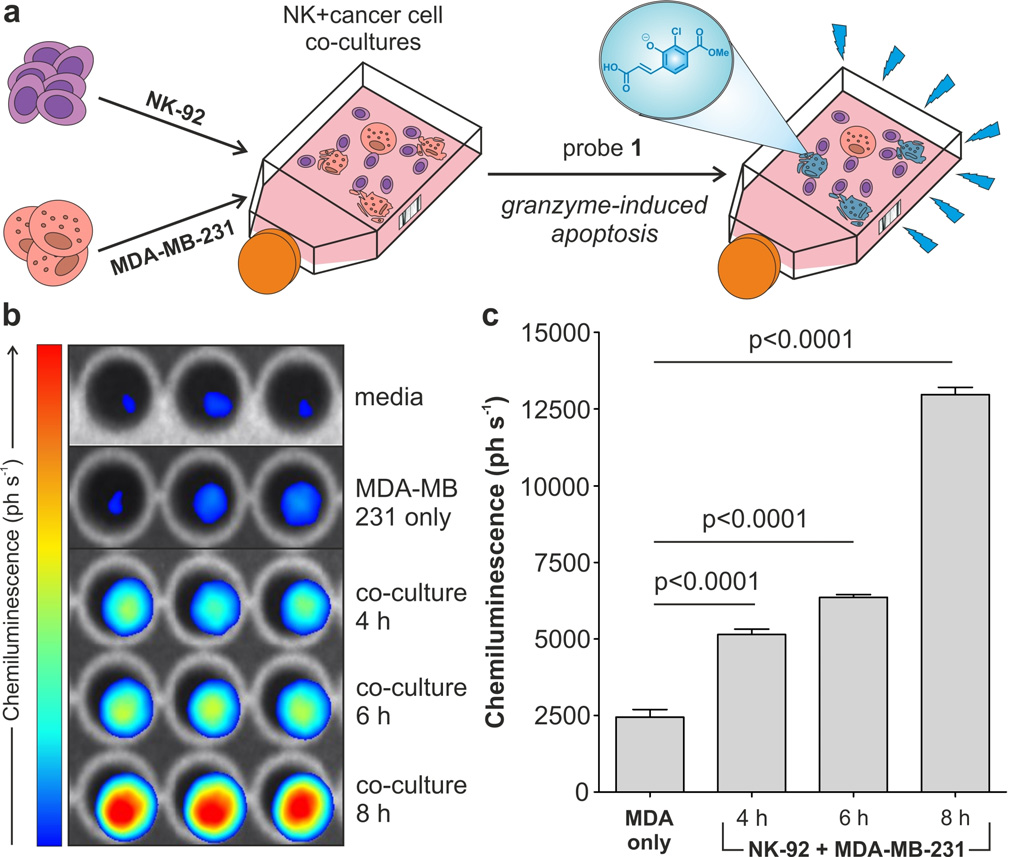
a) Representation of the co‐culture chemiluminescence assay to measure granzyme B‐mediated activity of NK cells against cancer cells. b) Chemiluminescence images of probe **1** (500 μm) incubated with either media only, MDA‐MB‐231 cells alone or co‐cultures of MDA‐MB‐231 cells NK‐92 cells for indicated times. c) Quantification of the chemiluminescence (in ph s^−1^) generated by probe **1** for different conditions. Data as means±SEM (*n*=3). P values from ONE‐ANOVA tests with multiple comparisons.

Finally, we examined the ability of probe **1** to image NK cell activity in vivo. We used a xenograft mouse cancer model where we injected immunocompromised NSG mice with human MDA‐MB‐231 cancer cells to grow subcutaneous tumours on both flanks of the animals. NSG mice lack host NK cells, thus representing an optimal model to assess the efficacy of adoptively transferred NK‐92 cells. First, MDA‐MB‐231 cells were injected to both flanks of the mice and grown for 3 weeks until two tumours (approx. 0.5–1 cm in diameter) were formed. At this point, NK‐92 cells cultured in the presence of IL‐2 (2×10^6^ cells/mouse) were injected into only one of the two tumours. Eight hours post NK cell transfer, probe **1** was administered to both tumours (i.e., tumours with and without NK cells) and whole‐body imaging was performed immediately in a PhotonImager^TM^ system. As shown in Figure 4, bright chemiluminescence signals were detected only in tumours that had received NK cell injection. Probe **1** showed good stability in serum (Supplementary Figure 9) and produced very low chemiluminescence in tumours without NK cells. Moreover, no signals were detected in mice that had not been injected with probe **1** (Figure 4). Tumours from NK‐treated mice were harvested and imaged ex vivo to confirm that the chemiluminescence signal of probe **1** was derived from the presence of active NK cells in tumours (Supplementary Figure 10). The results obtained from this study highlight that probe **1** acts as a first‐in‐class chemiluminescent probe to image NK cell activity in live tumours in vivo.

**Figure 4:**
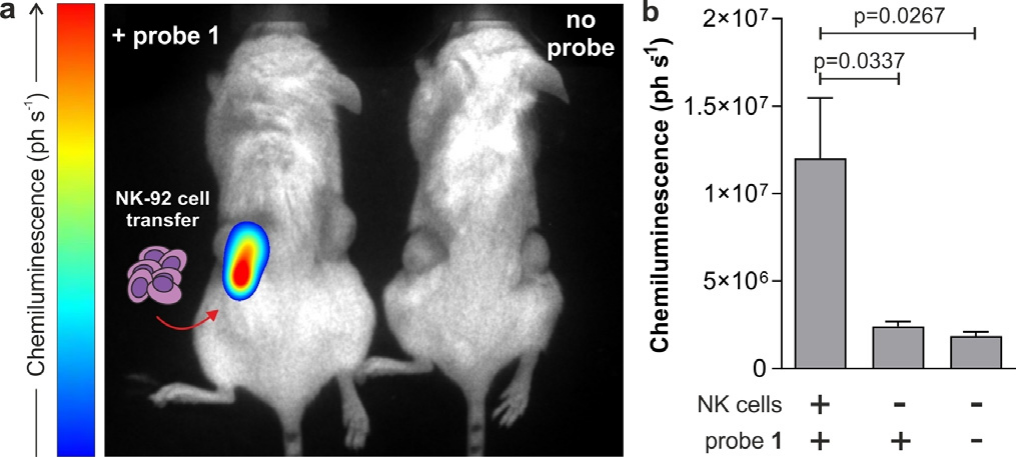
a) In vivo representative chemiluminescence images (from 3 independent experiments) of NSG mice containing MDA‐MB‐231‐xenograft tumours where NK‐92 cells were adoptively transferred. In all mice, MDA‐MB‐231 xenograft tumours were grown to 0.5–1 cm diameter in size. Left) only the right tumour (red arrow) was injected with NK‐92 cells (2×10^6^ cells/mouse) with the left tumour being NK cell‐free. After 8 h of the NK cell injection, probe **1** (125 μg) was injected to both tumours. Right) control animal with no injection of probe **1**. Whole‐body images were acquired immediately after injection of probe **1** and processed with M3Vision™ software. b) Quantification of chemiluminescence emission (ph s^−1^) for each condition displayed. Data as mean values±SEM (*n*=3). P values obtained from ONE‐ANOVA tests with multiple comparisons.

In summary, we have developed a new chemiluminescent probe for rapid and selective detection of NK cell activity in response to tumour cell recognition. We demonstrated that the activatable phenoxydioxetane reporter produces bright chemiluminescence after recognition by granzyme B but not with other closely related serine proteases. Probe **1** outperforms the commercial fluorogenic reagent Ac‐IEPD‐AMC in speed and signal‐to‐noise ratios and can be used to quantitatively measure NK cell function in co‐cultures of human NK cells and cancer cells. Furthermore, we have used probe **1** in a preclinical mouse model of NK cell adoptive transfer where tumours that had been recognised by NK cells were visualized in vivo using whole‐body chemiluminescence imaging. This study highlights the utility of activatable chemiluminescent probes to directly monitor the efficacy of NK cell‐based immunotherapies in live tumours.

## Conflict of interest

The authors declare no conflict of interest.

## Supporting information

As a service to our authors and readers, this journal provides supporting information supplied by the authors. Such materials are peer reviewed and may be re‐organized for online delivery, but are not copy‐edited or typeset. Technical support issues arising from supporting information (other than missing files) should be addressed to the authors.

Supplementary
